# Experiences With Maryland’s All-Payer Model Among Surgeons

**DOI:** 10.1001/jamanetworkopen.2025.52815

**Published:** 2026-01-07

**Authors:** Ronnie L. Shammas, Laura J. Fish, Laura A. Petrillo, Margaret Falkovic, Christina Makarushka, Heather Parnell, Amit Jain, Sheri S. Slezak, Aviram M. Giladi, Babak J. Mehrara, Nancy L. Keating, Evan Matros, Oluseyi Aliu, Anaeze C. Offodile

**Affiliations:** 1 Plastic and Reconstructive Surgery Service, Memorial Sloan Kettering Cancer Center, New York, New York; 2 Duke Cancer Institute Behavioral Health and Survey Research Core, Durham, North Carolina; 3 Division of Palliative Care and Geriatrics, Massachusetts General Hospital, Boston; 4 Department of Orthopaedic Surgery, Johns Hopkins University, Baltimore, Maryland; 5 Department of Surgery, University of Maryland School of Medicine, Baltimore; 6 The Curtis National Hand Center, MedStar Union Memorial Hospital, Baltimore, Maryland; 7 Department of Health Care Policy, Harvard Medical School, Boston, Massachusetts; 8 Division of General Internal Medicine, Brigham and Women’s Hospital, Boston, Massachusetts; 9 Division of Plastic and Hand Surgery, Department of Surgery, Allegheny Health Network, Pittsburgh, Pennsylvania

## Abstract

**Importance:**

Hospital global budgets gained attention as a strategy to constrain costs and improve outcomes. The Maryland all-payer model (APM), initiated in 2014 as a global budget revenue model, is an example of full-risk payment reform; yet frontline surgeon perspectives on the implementation and impact of this APM have not been evaluated.

**Objective:**

To examine surgeon experiences working under Maryland’s APM.

**Design, Setting, and Participants:**

This qualitative study used a convergent mixed-methods design to assess survey responses of surgeon experiences and semistructured interviews between June 15 and November 25, 2024. Maryland surgeons were recruited via purposive and snowball sampling for surveys from academic and community surgical practices. A nested sample of respondents was selected for qualitative interviews using maximum variation sampling.

**Main Outcomes and Measures:**

The primary outcome was surgeon-reported experiences with Maryland’s APM. Surveys and interview guides were designed using the Consolidated Framework for Implementation Research (CFIR) to assess awareness, communication, institutional engagement, changes in practice, and perceived effects on care delivery associated with the APM. Survey responses were summarized, and interview data were thematically analyzed and integrated using CFIR-guided joint displays.

**Results:**

Among 121 identified surgeons, 103 responded to the survey (67 [65.0%] male; practicing a mean [SD] of 16.4 [12.7] years), and 88 (85.4%) reported awareness of the APM. Of these 88 surgeons, a minority (35 [38.8%]) recalled information being distributed by their institution, and 41 (46.6%) reported receiving information from peers. Whereas 52 surgeons (59.1%) agreed that complex care had become more centralized, fewer believed that the model improved referral management (15 [17.0%] strongly agreed or agreed) or reduced preventable hospital use (16 [18.2%] strongly agreed or agreed). Most surgeons stated that the model changed the way they practiced at least slightly (56 [63.6%]). In qualitative interviews (n = 25), surgeons described surface-level understanding of the model and limited institutional communication, relying on peer discussions to interpret its implications. Many surgeons expressed confusion about performance expectations and frustration with the lack of feedback on quality metrics. They also noted that financial incentives shifted complex care to tertiary centers, contributing to resource strain.

**Conclusions and Relevance:**

In this mixed-methods study of surgeons’ experiences, surgeons reported high awareness of Maryland’s APM, but integrated findings revealed limited operational understanding, inconsistent institutional communication, and indirect effects on practice. Implementing alternative payment models may require more deliberate engagement of clinician stakeholders, clearer communication strategies, and alignment of clinical incentives.

## Key Points

**Question:**

What are the experiences and perspectives of surgeons practicing under Maryland’s all-payer model?

**Findings:**

This qualitative, mixed-methods study involving 88 surgeons found that there was high awareness of the model but limited understanding and inconsistent institutional communication about it. Surgeons acknowledged institutional efforts to improve outcomes but perceived that the model contributed to the centralization of complex care, imposed constraints on surgical practice, and felt disconnected from the development of quality initiatives intended to enhance patient care.

**Meaning:**

Findings suggest that implementing alternative payment models may require more deliberate engagement of clinician stakeholders, clearer communication strategies, and alignment of clinical incentives to ensure high-quality surgical care.

## Teaser

This qualitative, mixed-methods study investigates the experiences and perspectives of surgeons practicing under the Maryland all-payer model to assess model awareness, institutional communication and engagement, changes in practice, and perceived effects on care delivery associated with the model.

## Introduction

Surgical expenditures account for 7.3% of the United States’ gross domestic product and are growing rapidly.^1^ To address rising costs, the Centers for Medicare and Medicaid Services (CMS) and the state of Maryland introduced an all-payer model (APM) demonstration in 2014, beginning with the global budget revenue (GBR) model. This capped annual hospital revenue by prospectively setting a fixed budget for each hospital, encompassing most inpatient and outpatient services.^2^ Implementing the GBR constrained health care costs and improved patient outcomes at a population level.^3,4,5^ Based on this success, Maryland and CMS built on the APM by expanding Maryland’s GBR to a total cost of care (TCOC) model in 2019, which holds the state accountable for total health care spending across all payers (not just hospital costs).^6^

Under Maryland’s APM, hospital finances are influenced through penalties and rewards tied to performance metrics, such as readmissions and preventable complications.^6,7^ In a recent meta-analysis, implementation of the APM was associated with reductions in postsurgical complications, mortality, readmission, and length of hospital stay, while substantially reducing mean hospital costs.^5^ Additionally, Maryland’s APM was associated with spillover effects that altered the organization and provision of surgical care.^8,9^ Specifically, it was associated with the centralization of complex surgical care^9^ and accelerated shifts in elective surgical procedures to outpatient settings.^8^ These spillover effects likely influence surgeons’ experiences in providing care; however, to our knowledge, no study has evaluated surgeons’ perspectives on practicing under Maryland’s APM.

The CMS has signaled its commitment to full-risk capitated models by initiating the Achieving Healthcare Efficiency through Accountable Design model in Maryland, Connecticut, Hawaii, Vermont, and New York in 2024.^10^ As payment reform expands, understanding frontline surgeon experiences is critical to inform future policy and implementation strategies. In this study, we used a convergent mixed-methods design to explore surgeons’ experiences with Maryland’s APM—encompassing the GBR and TCOC initiatives—to identify key factors influencing surgical practice and understand whether spillover effects influenced surgeons’ experiences providing care.

## Methods

### Design and Rationale of Convergent Mixed-Methods Study

The survey and interview guide for this qualitative, mixed-methods study were designed using the Consolidated Framework for Implementation Research (CFIR). The study followed the Standards for Reporting Qualitative Research (SRQR) reporting guideline.^11,12,13^ The Memorial Sloan Kettering Institutional Review Board approved this study. Participants were not asked to distinguish between GBR and TCOC models but to reflect on their overall experience. Of note, the GBR is nested within the succeeding TCOC initiative.^14^ Quantitative and qualitative data were collected concurrently, analyzed separately, and then integrated (eFigure in Supplement 1).^15,16,17,18^ Data integration is reported through joint displays and in the discussion as meta-inferences.^16,18^Participants provided oral informed consent, as the study was conducted via videoconferencing calls.

### Study Population

We used snowball sampling to recruit surgeons in Maryland.^19^ Nonprobability sampling was chosen due to its effectiveness in reaching a specialized population (eg, surgeons).^20,21^ This aligned with the study’s aim to gather insights from surgeons familiar with the APM who could provide meaningful responses. Representatives were designated at Johns Hopkins Hospital (A.J.), University of Maryland Medical Center (S.S.S.), and MedStar Health (A.M.G.) to disseminate the survey and recruit participants. These entities represent Maryland’s 3 largest health care systems and account for over half of Maryland’s total hospital revenue.^22,23^ Surgeons were also recruited through the Maryland State Medical Society. For the qualitative investigation, we used maximum variation sampling to ensure diverse representation across surgical specialties, years in practice, and practice type.^24^ Participants were recruited from June 15 to November 25, 2024.

### Quantitative Data: Survey Development and Analysis Plan

We designed the survey according to CFIR domains: intervention characteristics, outer setting, inner setting, characteristics of individuals, and implementation process (eMethods 1 in Supplement 1).^25^ A 5-point Likert scale assessed agreement, ranging from 1 (strongly agree) to 5 (strongly disagree). Respondents self-reported demographics, including race and ethnicity, which were assessed to more fully characterize the cohort. The responses for the race and ethnicity followed the US Census guidelines for race and ethnicity and included Asian, Black or African American, Native Hawaiian or Other Pacific Islander, White, prefer not to answer, more than 1 race, and other, which included respondents identifying as a race not listed. We pilot-tested the survey with a convenience sample of 5 surgeons and made minor changes. The final survey comprised 23 items and was administered via Qualtrics (Qualtrics XM) through departmental listservs and Maryland State Medical Society newsletters. Quantitative data were summarized using frequencies, proportions, and means.

### Qualitative Data: Interview Design and Analysis Plan

We developed a semistructured interview guide according to CFIR. The guide included parallel questions to the survey.^25,26^ This parallel design allowed for the quantitative and qualitative datasets to be merged. The interview guide (eMethods 2 in Supplement 1) was pilot-tested with the same 5 surgeons; because only minor revisions were made, these interviews were included in the analysis. Interviews were conducted via videoconferencing. After every 5 interviews, transcripts were reviewed to assess information power, a pragmatic model guiding sample size determination in qualitative research.^27^ All interviews were recorded, transcribed verbatim, and analyzed using NVivo, version 14 (QSR International). Three investigators independently coded interview transcripts. The codebook is presented in eMethods 3 in Supplement 1.

## Results

### Study Participants Characteristics

Of 121 surgeons identified through snowball sampling who received the survey, 103 responded. Of those, 33 (32.0%) identified as female, 67 (65.0%) identified as male, 15 [14.6%] were Asian, and 72 (69.9%) were White. Respondents were practicing in Maryland for a mean (SD) of 16.4 (12.7) years. The most represented specialties were orthopedic (17 [16.5%]), vascular (12 [11.7%]), cardiothoracic (11 [10.7%]), and general (11 [10.7%]) surgery. Most respondents practiced in an academic setting (62 [60.2%]) (eTable in Supplement 1).

### Quantitative Survey Results

#### Intervention Characteristics: Awareness and Understanding

**Figure 1.**
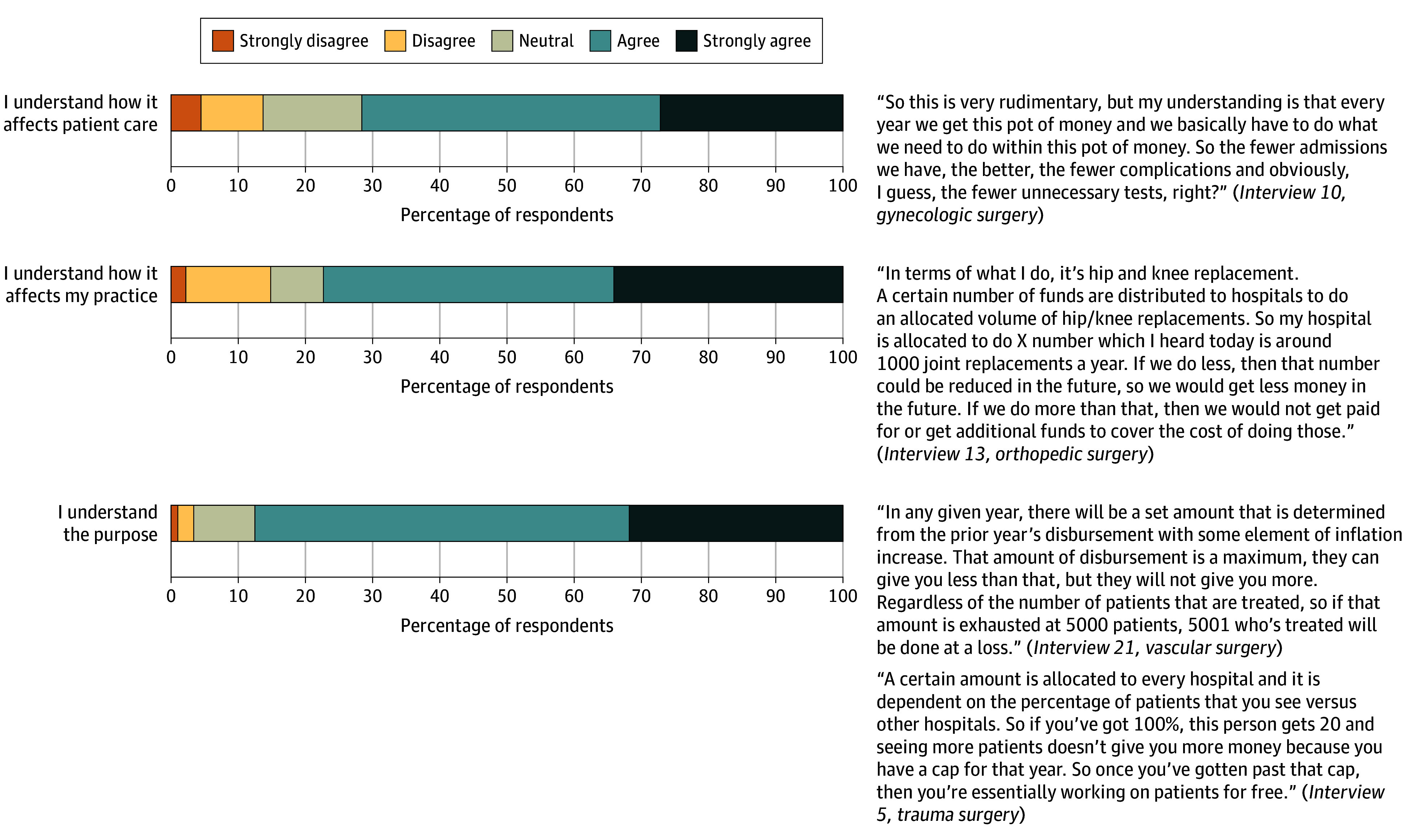
Intervention Characteristics: Perceived Understanding of the All-Payer Model

Among respondents, 88 (85.4%) reported being aware of the APM and proceeded with the survey. Those unaware of the model answered only demographic questions. Nearly all respondents strongly agreed or agreed that they understood how the APM affected patient care (63 [71.5%]), their practice (68 [77.2%]), and the purpose (77 [87.5%]) (Figure 1).

#### Implementation Process: Communication About the APM

**Figure 2.**
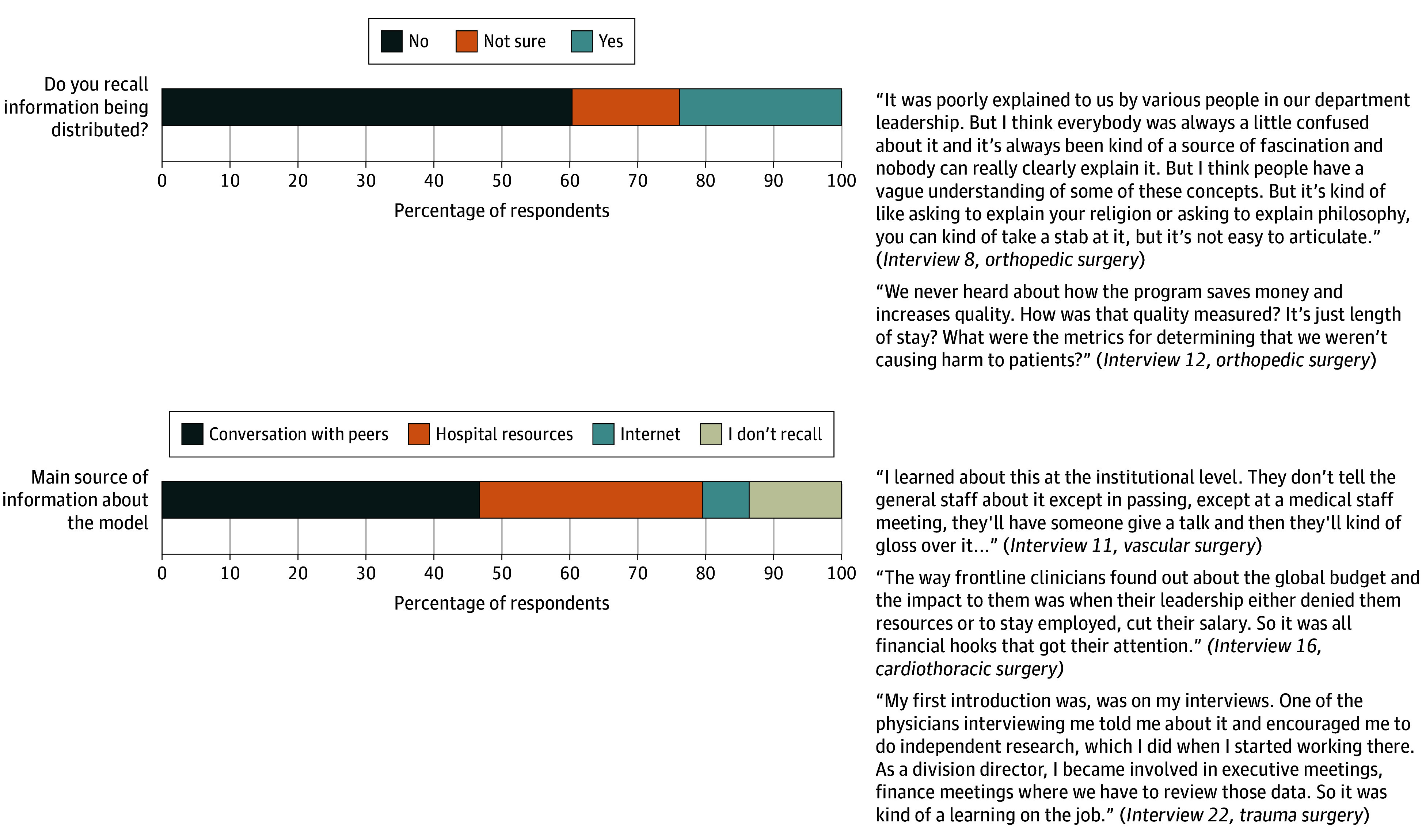
Implementation Process: Surgeon Perceptions on Communication

When asked how information about the model was communicated, a minority (35 [38.8%]) recalled receiving information from the hospital, practice, or health care system (Figure 2). When asked about their primary source of information, a plurality said it came from informal conversations with peers (41 [46.6%]), and only 29 (32.9%) recalled receiving information through hospital resources.

#### Outer Setting: Perceived Effect on Referral Networks, Regionalization, and Preventable Hospital Use

**Figure 3.**
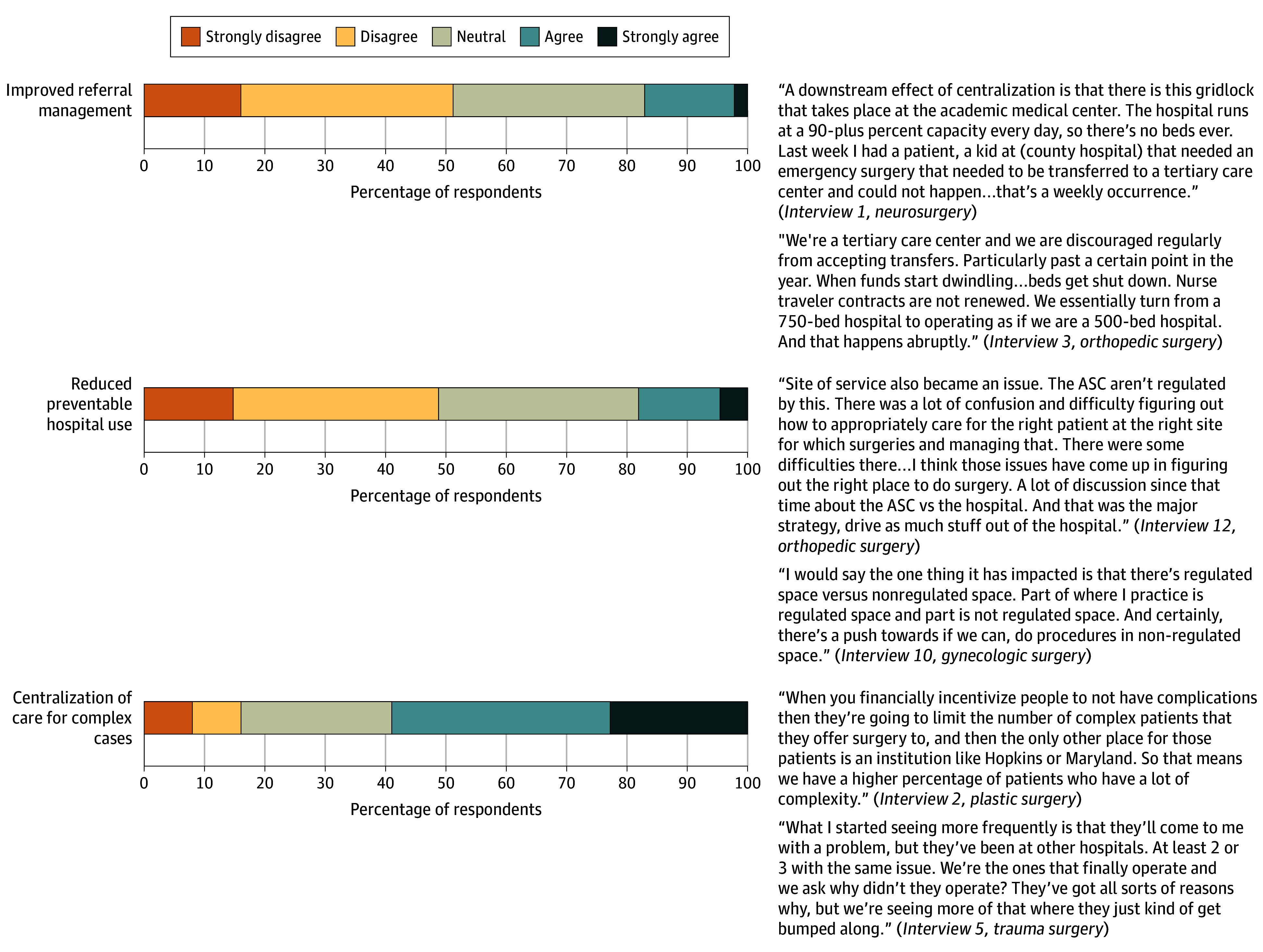
Outer Setting: Perceived Effect on Referral Networks, Regionalization, and Preventable Hospital Use ASC indicates ambulatory surgical center.

Roughly half of respondents strongly disagreed or disagreed that the APM improved referral management (45 [51.1%] vs 15 [17.0%] who strongly agreed or agreed), defined as the coordination and efficiency of directing patients to appropriate health care professionals or care settings, and reduced preventable hospital use (43 [48.9%] vs 16 [18.2%] who strongly agreed or agreed) (Figure 3). Most respondents strongly agreed or agreed that implementation resulted in the centralization of complex surgical care (52 [59.1%]).

#### Inner Setting: Surgeon Perceptions of Institutional Efforts to Enhance Patient Care

**Figure 4.**
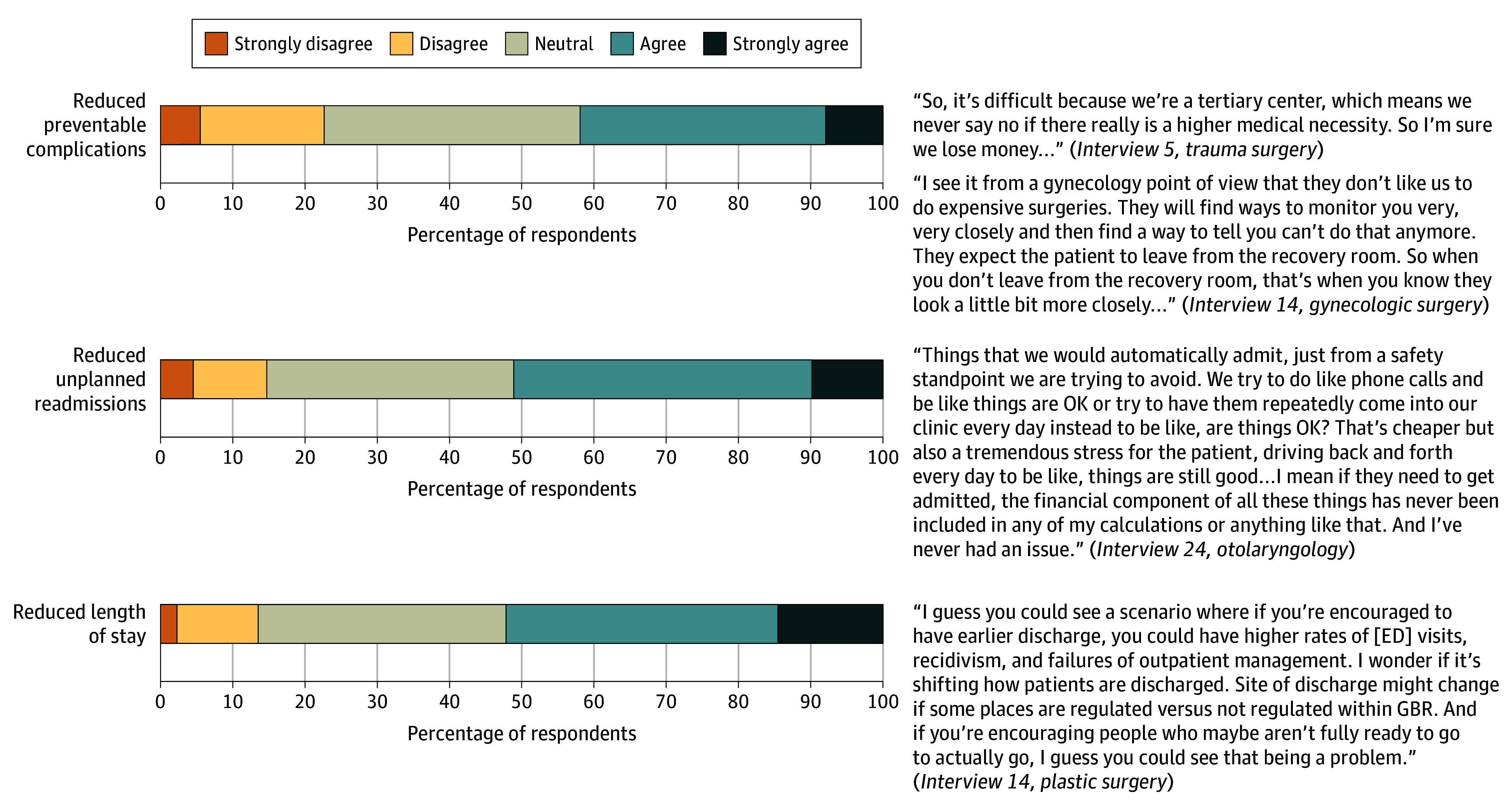
Inner Setting: Surgeon Perceptions of Institutional Efforts to Enhance Patient Care ED indicates emergency department; GBR, global budget revenue.

Fewer than half of respondents (37 [42.0%]) strongly agreed or agreed that there was a perceptible effort to reduce complications after implementation of the APM (Figure 4). Approximately half strongly agreed or agreed that there were efforts to reduce unplanned readmissions (45 [51.1%]) and length of hospital stay (46 [52.3%]).

#### Individual Characteristics: Perceived Influence on Surgeon Practice and Patient Care

**Figure 5.**
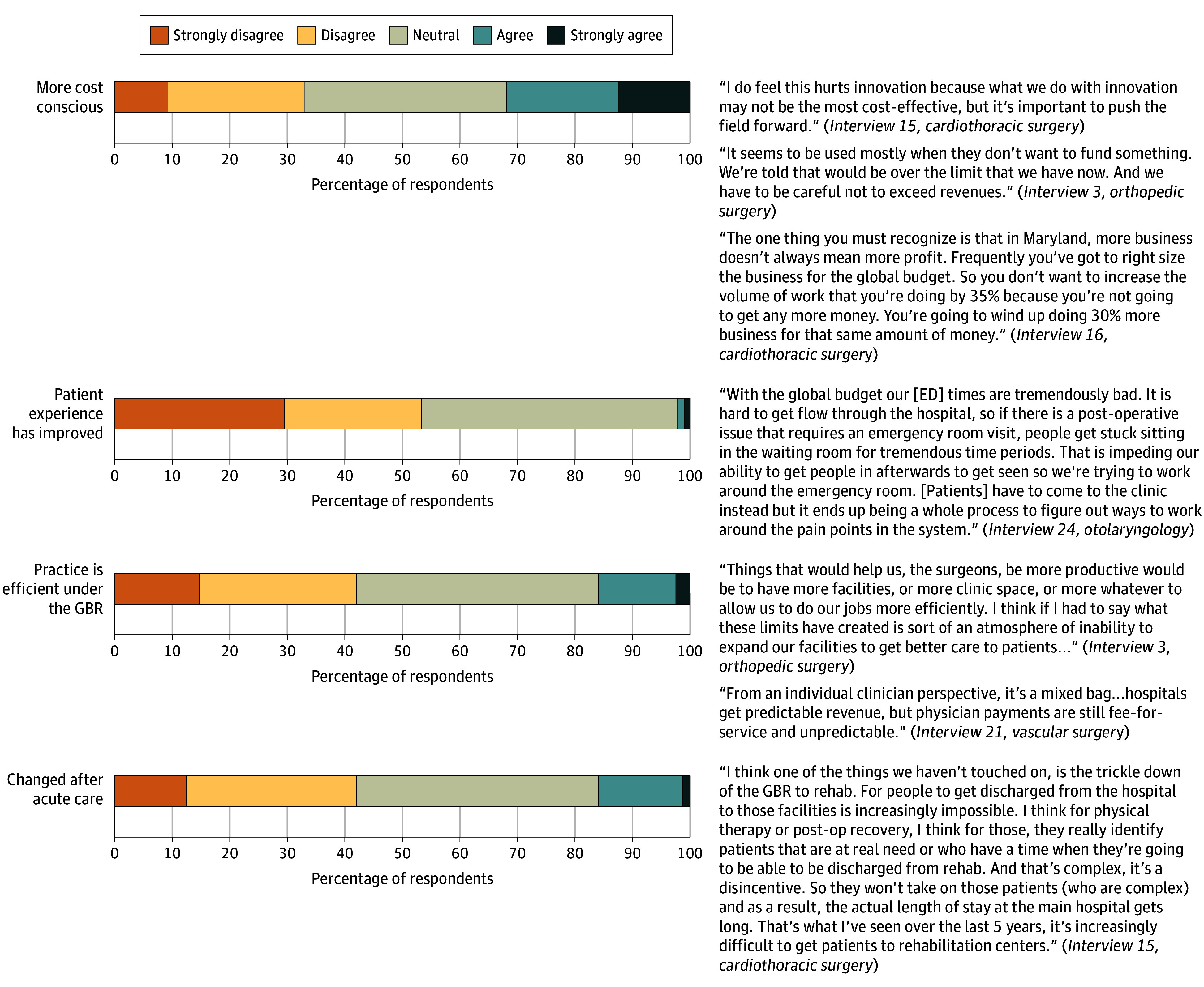
Individual Characteristics: Influence on Surgeon Practice and Patient Care ED indicates emergency department; GBR, global budget revenue.

When asked if implementing the APM made them more cost-conscious, many respondents reported being neutral (31 [35.2%]) (Figure 5). Most respondents strongly disagreed or disagreed that the APM improved patient experience (47 [53.4%]). When asked if the APM made their practice more efficient or changed their own postacute care utilization, 37 respondents (42.0%) strongly disagreed or disagreed. Most respondents stated that the APM changed their practice at least slightly (56 [63.6%]).

### Qualitative Findings

We conducted 25 semistructured interviews, each a mean (SD) of 32.9 (9.1) minutes. Qualitative findings were organized by CFIR domains and are presented with quantitative findings as joint displays in Figures 1 to 5.

#### Intervention Characteristics: Awareness and Understanding

Surgeons varied in their understanding of the APM, ranging from in-depth to superficial. Surgeons interested in payer systems and those in administrative roles were more aware of the APM. Surgeons generally understood that hospitals receive a predetermined annual revenue to cover all services, regardless of volume, and that hospitals face penalties if they exceed their budget. Surgeons who practiced solely in an inpatient setting were more likely to report that the APM did not impact their practice. Thus, they did not have as much awareness as surgeons who also operated in an outpatient setting.

#### Implementation Process: Communication About the APM

Surgeons expressed frustration regarding the quality and frequency of institutional communication. Many indicated receiving minimal or no formal communication from their hospitals or health care systems, describing information dissemination as fragmented and inadequate. Consequently, surgeons reported relying heavily on peer interactions and casual discussions to understand the model and its impact on their practice. This absence of institutional communication led to inconsistent knowledge sharing, confusion, and uncertainty among surgeons about expectations, evaluation metrics, and the model’s influence on clinical workflow. Surgeons emphasized that these communication gaps negatively influenced their working experience and perceived that there was no coordinated effort to provide information to clinicians or incorporate surgeon input in the model’s design.

#### Outer Setting: Perceived Effect on Referral Networks, Regionalization, and Preventable Hospital Use

Many surgeons described an increased centralization of complex procedures due to financial pressures discouraging community hospitals from investing in high-cost specialty services. Surgeons at tertiary centers noted increased transfer requests for complex or resource-intensive surgical patients, creating perceived strain on bed availability and operative scheduling. Surgeons reported that this increased volume resulted in a bottleneck, contributing to delays for emergent and nonemergent transfers. Many surgeons perceived little to no meaningful improvement in reducing preventable hospital use or managing referrals, citing ongoing logistical challenges and inefficiencies in patient flow management. Surgeons expressed concern that centralization could limit timely access to care and negatively influence outcomes.

#### Inner Setting: Surgeon Perceptions of Institutional Efforts to Enhance Patient Care

While some surgeons described an emphasis from hospital leadership on improving outcomes (readmissions, length of hospital stay, preventable complications), others said that these efforts were inconsistently communicated. Some cited institutional investments in care coordination, case management, and quality reporting. However, many surgeons perceived that frontline clinicians’ engagement was limited, with few opportunities to contribute to quality initiatives or receive actionable performance feedback.

#### Individual Characteristics: Perceived Influence on Surgeon Practice and Patient Care

While some surgeons noted increased awareness of hospital-level metrics (eg, readmissions and complications), most reported that these factors had limited influence on their clinical decision-making. Several surgeons highlighted that individual reimbursement remained tied to fee-for-service models. Surgeons discussed the broader effects of the model on their working environment, citing limitations on programmatic and service line growth and constrained access to new equipment. Many noted that this limited-growth mindset contributed to diminished patient experiences and difficulties with surgeon recruitment and retention. Several surgeons noted a shift of lower-complexity procedures to ambulatory surgical centers to reduce inpatient volume and avoid budget penalties. However, limitations in access and institutional reluctance to expand outpatient capacity constrained this shift.

## Discussion

In this qualitative, mixed-methods study, we explored the experiences of surgeons working under Maryland’s TCOC model, many of whom also practiced under the GBR model between 2014 and 2018. We identified key themes related to awareness, communication, institutional engagement, and changes in clinical practice. Our findings suggested that while surgeons were broadly aware of the APM’s cost-containment goals, substantial gaps remained in institutional communication, incentive alignment, and support for clinician engagement. Policymakers may perceive a limited need to engage surgeons in payment reform; however, institutional incentives under the APM indirectly shape surgical delivery. Reasons to engage surgeons are to ensure the sustainability of quality gains and cost savings and to mitigate and solve unintended consequences (eg, centralization, site of care optimization). In addition, expanding beyond inpatient cost containment to outpatient, primary, and population health domains requires deeper clinical integration, and surgeons will be essential in redesigning delivery models that achieve these broader goals.

### Integrated Findings

#### Awareness and Understanding

Surgeons reported broad awareness of the APM, with most indicating familiarity and agreeing that they understood its purpose. However, qualitative data revealed this understanding was surface level, particularly among surgeons without administrative roles, who described their knowledge as “cursory” or “general” and had a limited grasp of operational details. These convergent findings support the meta-inference that while most surgeons are aware of the APM, many lack the understanding to meaningfully engage with or respond to its goals. Kilaru et al^12^ similarly reported that health care leaders perceived a disconnect between frontline clinicians and the implementation goals of the Maryland APM. Friedberg et al^28,29^ also noted that complexity and limited transparency in alternative payment models may contribute to confusion among physicians, even in systems with high awareness.

#### Communication

Surgeons reported limited institutional communication regarding the APM, with most stating that they did not receive formal information. Instead, nearly half of surgeons reported learning about the APM informally through conversations with peers. Qualitative findings echoed this, with many surgeons describing the absence of structured communication or educational efforts and expressing frustration about the lack of clear messaging on how the model affects their practice. Despite the model’s long-standing implementation, these convergent data support the meta-inference that poor communication infrastructure has contributed to uneven awareness and limited clinician engagement. Improving communication strategies is essential to fostering accountability within future redesign initiatives, especially as these reform efforts expand beyond inpatient hospital care to encompass outpatient and primary care services.^10^ Without deliberate efforts to inform clinicians, the lack of structured communication may persist as a barrier to health care professional engagement and the successful adoption of population-based payment reform.

#### Effect on Centralization and Referrals

Most surgeons agreed that complex surgical cases had become more centralized, while fewer surgeons believed that the APM improved referral management or reduced preventable hospital use. Qualitative findings reinforced this, with surgeons describing increased transfer requests to tertiary centers and increasing bottlenecks in surgical scheduling and inpatient capacity. These convergent results suggest that the APM may have disincentivized community hospitals from managing high-acuity, high-cost patients.^9^ Under the APM, hospitals are penalized for exceeding their budget and for quality metrics, which may lead hospitals to refer complex cases to tertiary centers better equipped to absorb these risks. Prior studies have similarly identified centralization of surgical care as a spillover effect.^9^ As APMs evolve to cover a broader range of services, careful attention will be needed to ensure that incentives designed to contain costs do not unintentionally discourage the provision of complex surgical care or contribute to access limitations.^30^

#### Institutional Efforts to Enhance Patient Care

Approximately half of the respondents agreed that their institutions made efforts to reduce readmissions, length of hospital stay, and preventable complications. Qualitative data revealed that while some surgeons knew of investments in care coordination or quality improvement, many described limited frontline involvement and a lack of direct feedback on performance metrics. These convergent findings suggest that institutional quality improvement efforts may not consistently reach or engage surgeons, despite organizational accountability under the APM. This disconnect may weaken opportunities to align clinical behavior with institutional goals and undercut the potential for more coordinated improvements in surgical care. Similar findings have been reported in broader evaluations of alternative payment models, in which limited clinician engagement has been cited as a key implementation barrier, particularly when communication and feedback systems are insufficient to support shared accountability.^28^

#### Surgeon Practice and Patient Care

Most surgeons acknowledged that the model changed their practice; however, few felt it improved efficiency or patient experience. Qualitative data revealed that although some surgeons had heightened awareness of performance metrics, many noted that their compensation remained tied to fee-for-service reimbursement, limiting the model’s direct influence on their decision-making. Additionally, surgeons described structural impacts of the APM, including constrained resources, limitations on new programs, and reduced inpatient operating room access, which they perceived as negatively affecting innovation, care delivery, and recruitment. Our qualitative findings, wherein surgeons reported a shift in elective procedures to the ambulatory setting, are also supported by a recent analysis.^8^ These findings support the meta-inference that while the APM shapes institutional priorities, it also indirectly influences individual surgeon practice, which can lead to frustration and confusion in the absence of aligned financial incentives. Prior research has similarly shown that physicians participating in alternative payment models experience increased institutional pressure without corresponding compensation changes or structural support, which may contribute to frustration, disengagement, or resistance to reform.^28^

#### Recommendations

Experiences from episode-based payment models indicate that surgeon engagement is most effective when hospitals operationalize clear incentives, data, and governance at the service-line level. Under the Comprehensive Joint Replacement Program, CMS permitted hospitals to share reconciliation payments with physicians through gainsharing, creating direct alignment between clinical practice and model performance.^31^ Hospitals participating in joint-replacement bundles reduced spending and postacute care through care-pathway standardization and discharge planning, changes that depend on surgeon leadership. Similarly, when physician groups have led or co-led bundled care program participation, episode performance was comparable with or better than hospital-led participation, highlighting the value of clinician-directed redesign and feedback loops.^32^ Limited surgeon engagement in Maryland’s APM mirrors broader patterns observed across payment reform efforts, in which physician involvement is often variable and concentrated among early adopters.^32,33^ Rather than aiming for universal participation, payment models should prioritize structured, role-appropriate engagement. Offering practice transformation support (eg, data analytics and real-time dashboards for tracking complications, operating room efficiency, and disposable supply costs) directly to surgeons can increase awareness and align their behavior and clinical practice with the goals of the APM.^34^ Consistent with CMS guidance on specialty engagement,^35^ we recommend that health systems support physician engagement by (1) providing structured surgeon onboarding and health care professional–level dashboards with performance feedback, (2) establishing joint governance (eg, steering committees) to codesign pathways and referral criteria, (3) implementing internal gainsharing, and (4) allocating protected time and support to execute changes.

### Limitations

This study has limitations. Our sampling imbalance of academic surgeons may have influenced our findings in several ways: community surgeons may have different experiences, particularly regarding institutional communication, engagement in quality initiatives, and exposure to centralized complex care; and academic surgeons may be more attuned to the spillover effects of the APM, such as the centralization of high-acuity care. Other specialties, such as primary care physicians and hospitalists, are more likely to be influenced by hospital-level metrics of the APM (eg, readmissions, hospital-acquired conditions, emergency department visits, and avoidable utilization). Future work should intentionally engage a broader sample of community and private practice health care professionals to fully understand the impact of the APM across the broader health care professional landscape.

## Conclusions

This qualitative, mixed-methods study found that while awareness among surgeons of the Maryland APM was high, most had a limited operational understanding, partly due to inconsistent institutional communication. Surgeons reported that the APM contributed to the regionalization of complex care, impacted institutional resources, and led to institutional constraints on surgical practice but had limited influence on clinical decision-making. Future research should assess community surgeon engagement, identify strategies to enhance and sustain surgeon involvement, and examine the effect of the APMs on patient access and health equity.

## Notes

Supplement 1. **eFigure.** Convergent Mixed-Methods Study Design **eMethods 1.** Survey Questions Organized by CFIR Domains **eMethods 2.** Interview Guide **eMethods 3.** Qualitative Codebook **eTable.** Demographic and Occupational Characteristics of Respondents

Supplement 2. Data Sharing Statement
