# Supplementary material for: Experiences With Maryland’s All-Payer Model Among Surgeons
Source: JAMA Netw Open. 2026 Jan 7;9(1):e2552815. doi: 10.1001/jamanetworkopen.2025.52815 (PMC12780931; doi:10.1001/jamanetworkopen.2025.52815)
Supplement: Supplement 1. — eFigure. Convergent Mixed-Methods Study Design eMethods 1. Survey Questions Organized by CFIR Domains eMethods 2. Interview Guide eMethods 3. Qualitative Codebook eTable. Demographic and Occupational Characteristics of Respondents [file jamanetwopen-e2552815-s001.pdf]

Supplementary Online Content

Shammas RL, Fish LJ, Petrillo LA, et al. Experiences with Maryland's all-payer model among surgeons. *JAMA Netw Open*. 2026;9(1):e2552815. doi:10.1001/jamanetworkopen.2025.52815

eFigure. Convergent Mixed-Methods Study Design

eMethods 1. Survey Questions Organized by CFIR Domains

eMethods 2. Interview Guide

eMethods 3. Qualitative Codebook

eTable. Demographic and Occupational Characteristics of Respondents

This supplementary material has been provided by the authors to give readers additional information about their work.

© 2026 Shammas RL et al. *JAMA Network Open*.

eFigure. Convergent Mixed-Methods Study Design

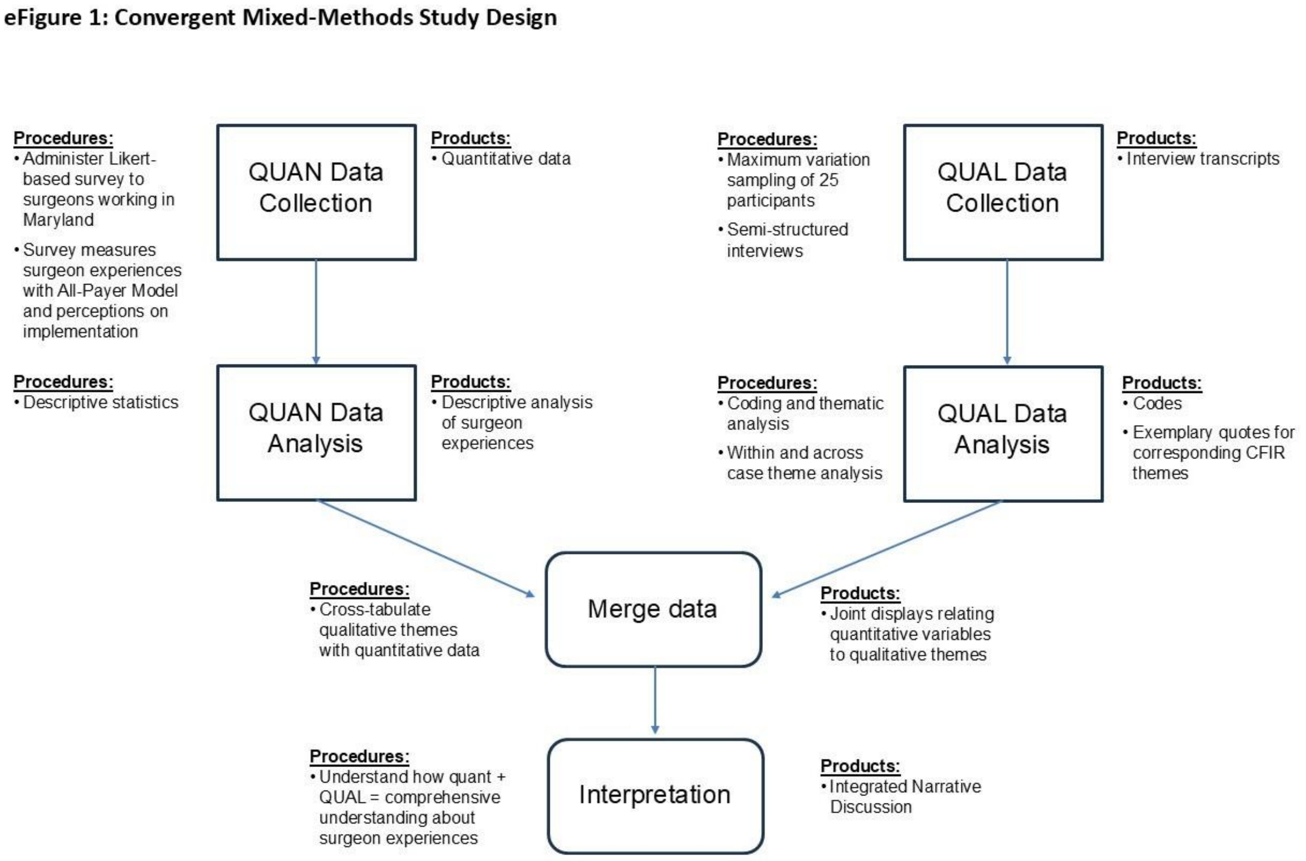

© 2026 Shammas RL et al. *JAMA Network Open*.

eMethods 1. Survey Questions Organized by CFIR Domains

**Intervention Characteristics**
**Are you aware of the Maryland Global Budget model?**
**I understand the purpose of the Maryland Global Budget model.**
**I understand how the Maryland Global Budget model affects my practice.**
**I understand how the Maryland Global Budget model affects patient care.**
**Implementation process**
**Do you recall that information about the Maryland Global Budget model was distributed to clinicians in your health system?**
**What was the main source of your information about the Maryland Global Budget model?**
**How satisfied were you with this communication?**
**Outer Setting**
**The Maryland Global Budget model has improved referral management.**
**I feel that under the Maryland Global Budget model, complex cases are increasingly shifted to high-volume centers.**
**Under the Maryland Global Budget model, hospitals have focused more resources on reducing preventable hospital use.**
**Inner Setting**
**Under the Maryland Global Budget model, hospitals have focused more resources on reducing the length of stay after surgery.**
**Under the Maryland Global Budget model, hospitals have focused more resources on reducing unplanned readmission and/or emergency department visits after surgery.**
**The Maryland Global Budget model has reduced preventable complications.**
**Characteristics of Individuals**
**I feel that my practice is efficient under the Maryland Global Budget model.**
**I think that my patients' experience with care has improved since implementation of this model.**
**Since implementation of the Maryland Global Budget model, I am more conscious of the costs of care for my patients.**
**The Maryland Global Budget model has changed the way I practice.**
**Under the Maryland Global Budget model, I have changed how I select post-acute settings of care.**

© 2026 Shammas RL et al. *JAMA Network Open*.

eMethods 2. Interview Guide

The Maryland Global Budget model, launched in 2014, established global budgets for certain Maryland hospitals to reduce Medicare hospital expenditures and improve quality of care for beneficiaries. Global budgets provide hospitals with a fixed amount of revenue for the upcoming year. A global budget encourages hospitals to eliminate unnecessary hospitalizations, among other benefits. Under the Global Budget model, Maryland achieved significant savings for Medicare and improved quality.

The goal of this interview is to hear your experience working and caring for patients as a surgeon working under the Global Budget model. There are no right or wrong answers to the questions we ask. Your responses will be recorded and analyzed to assess what lessons can be learned from your experience and how we may use this to improve health care redesign efforts in the future.

1. **Please tell me about the scope of your practice and your role at your institution.**

I am interested in hearing about your experience as a surgeon treating patients under Maryland's Global Budget model and how you feel this may ultimately impact patient care.

2. **In your own words, can you explain to us what your understanding is of the Maryland Global Budget model?**

   – What was your understanding of how the global budget model was rolled out from the state level?

3. **Please tell me about what information was delivered to you about this model and its continued implementation in Maryland.**

   – How did you learn about the Maryland Global Budget model?
     Probes: how was information relayed to you? (i.e. emails, journal articles, etc.)

   – When was this information delivered and how often?

4. **How was the Maryland Global Budget model introduced to you by the hospital or practice you work in?**

   - What was your understanding of the goals for implementation at your hospital or practice?
   - How and when were updates delivered to you on the outcomes of this healthcare reform at your hospital or practice?
   - What communication did you receive on how this would directly impact surgical practice and procedures?

5. **How do you feel that the global budget model influences your practice/institution?**

   – How has it impacted referral patterns?

   – How has it impacted your practice environment? (i.e. ratio of elective or outpatient procedures)

   – How has this impacted care for more complex surgical procedures (i.e. Centralization of complex surgical procedures)?

© 2026 Shammas RL et al. *JAMA Network Open*.

– What are things that you think went well or poorly with the implementation of the global budget model?

– What / if any roadblocks did you experience with patient care as a result of the introduction of the model?

6. **Given what you understand about the global budget what would define success of this program?**

   – How would your definition of success differ from that of the institution or the state?

7. **Given what you understand about the global budget, what have been key barriers to introducing the Maryland Global Budget model to your practice/institution?**

   – Prompt: Information delivery

8. **Given what you understand about the global budget, have been key facilitators to introducing the Maryland Global Budget model to your practice?**

   – Prompt: Information delivery

9. **Given what you understand about the global budget, what are unintended consequences of this program that you think could arise and influence patient care or the patient experience?**

   – Prompts:
   - Wait time
   - Travel time
   - Complications
   - Length of stay

10. **In a perfect world, what would be the best way to control health care costs related to surgery?**

11. **What else is important for us to know about surgeons and their experience with the Global Budget model in Maryland?**

© 2026 Shammas RL et al. *JAMA Network Open*.

eMethods 3. Qualitative Codebook

**Scope of practice and role**	Participants describe their practice (academic, hospital-based, private practice, combination and their role in their practice.<br><br>Include any discussion of a leadership role in the practice/institution
**Understanding of Maryland APM**	Participants' understanding of what APM is and what it was developed to do for healthcare costs.
**Learning about APM**<br>**Method and reaction**	Discussion of how APM was introduced to their practice or institution or department and how they receive ongoing information about APM.<br>• Include discussion of participants engaged in self-directed research and education about APM.<br>• Understanding the goals at their institution;<br>• Discussion or lack of discussion of any communication about APM from the department or from the institution.
**Influence on practice**	Discussion of positive and negative impact of APM on the provider's practice and institution. This includes the impact of APM on<br>• Impact on surgeon decision-making about patients<br>• Referral process and conducting complex surgical procedures.<br>• How cases are scheduled across surgery locations (Specifically, funneling of less complex cases to ambulatory surgery centers, and more complex cases funneled to major hospitals).<br>• How care is delivered (i.e. telemedicine)<br>• Impact on physician salaries/reimbursement.<br>• How hospitals may be penalized for not meeting certain criteria (thresholds for length of stay, readmissions, complications).<br>• How different health settings are affected by APM<br>• Impact on hiring and retention of surgeons and other staff in the hospital.
**Defining success**	Participants perspective on what defines success when working to control cost through the APM
**Barriers to implementation**	Factors that have hindered or been challenging with implementation of APM at the patient, provider, practice, institution, or policy level.<br>• Things that went poorly
**Facilitators to implementation**	Factors that have supported or helped with implementation of APM at the patient, practice, institution, or policy level.<br>• This includes communication about the APM<br>• Things that went well
**Unintended consequences for patients**	Discussion of the impact of APM on patient experience.<br>• Including length of stay impacted by the availability of beds in SNF,<br>• increased wait times for surgery,

© 2026 Shammas RL et al. *JAMA Network Open*.

	• longer distance to travel,<br>• increased complications after surgery<br>• Positive impact
**Recommendations for controlling cost**	Participants' opinions about how to control healthcare costs in an ideal world
**Other**	Any other important things the participant discussed.<br>Looking to the future - concern about APM impact on utilization as elderly population increases
**Interesting**	Use this code for something that stands out to you or something that may need its only own code

© 2026 Shammas RL et al. *JAMA Network Open*.

eTable. Demographic and Occupational Characteristics of Respondents

Characteristic	Survey Respondents, No (%) (n = 103)
**Gender**	
Male	67 (65)
Female	33 (32)
Prefer not to say	3 (2.9)
**Race**	
Asian	15 (14.6)
Black or African American	4 (3.9)
Native Hawaiian or Other Pacific Islander	1 (1)
White	72 (69.9)
I would prefer not to answer	5 (4.9)
More than one race	5 (4.9)
Other*	1 (1)
**Ethnicity**	
Hispanic/Latino	3 (2.9)
Not Hispanic/Latino	100 (97.1)
**Years in practice, average (SD)**	19.71 (12.68)
**Years practicing in Maryland, average (SD)**	16.4 (12.46)
**Surgical Specialty**	
Orthopedic	17 (16.5)
Vascular	12 (11.7)
Cardiothoracic	11 (10.7)
General	11 (10.7)
Otolaryngology	9 (8.7)
Obstetrics and Gynecology	9 (8.7)
Trauma	9 (8.7)
Plastic	8 (7.7)
Ophthalmologic Surgery	5 (4.9)
Urology	4 (3.9)
Pediatric	3 (2.9)
Colorectal	2 (1.9)
Other	3 (2.9)
**Practice Type**	
Academic	62 (60.2)
Private Practice	17 (16.5)
Hospital Employed	16 (15.5)
Hybrid Academic/Private Practice	7 (6.8)
Other	1 (1)
**Average (SD) percent time spent with:**	
Clinical	76.68 (17.36)
Administrative	14.78 (13.33)
Research	6.77 (10.82)
Other	1.87 (6.32)

*Other includes respondents identifying as a race not listed among the listed categories

© 2026 Shammas RL et al. *JAMA Network Open*.
